# Duplex ultrasonography for the detection of vertebral artery stenosis: A comparison with CT angiography

**DOI:** 10.1002/brb3.750

**Published:** 2017-06-29

**Authors:** Anouk D. Rozeman, Hajo Hund, Michel Westein, Marieke J. H. Wermer, Geert J. Lycklama à Nijeholt, Jelis Boiten, Robert‐Jan Schimsheimer, Ale Algra

**Affiliations:** ^1^ Department of Neurology MCHaaglanden The Hague The Netherlands; ^2^ Department of Radiology MCHaaglanden The Hague The Netherlands; ^3^ Department of Clinical Neurophysiology MCHaaglanden The Hague The Netherlands; ^4^ Department of Neurology LUMC Leiden The Netherlands; ^5^ Department of Clinical Epidemiology Leiden The Netherlands; ^6^ Department of Neurology and Neurosurgery Brain Center Rudolf Magnus UMC Utrecht Utrecht The Netherlands

**Keywords:** CT angiography, duplex ultrasonography, vertebral artery, vertebrobasilar stroke

## Abstract

**Objectives:**

Vertebrobasilar stenosis is frequent in patients with posterior circulation stroke and it increases risk of recurrence. We investigated feasibility of duplex ultrasonography (DUS) for screening for extracranial vertebral artery stenosis and compared it with CT angiography (CTA).

**Materials and Methods:**

We gathered data on 337 consecutive patients who had DUS because of posterior circulation stroke or TIA. Matching CTA studies were retrieved and used as reference. Stenosis on CTA was considered “significant” if >50%, at DUS if Peak Systolic Velocity (PSV) > 140 cm/s for the V1 segment and PSV > 125 cm/s for the V2 segment. We determined the area under the ROC curve (AUROC). In addition, we calculated which PSV cut‐off value resulted in highest sensitivity with acceptable specificity.

**Results:**

DUS was able to make an adequate measurement in 378 of 674 V1 segments and 673 of 674 V2 segments. DUS detected a significant stenosis in 52 of 378 V1 segments; 12 were confirmed by CTA (AUROC 0.73, 95% Confidence Interval 0.63–0.83). The optimal DUS PSV cut‐off value for this segment was 90 cm/s. For the V2 segment there were too few stenoses to allow reliable assessment of diagnostic characteristics of DUS.

**Conclusions:**

Although DUS has a fair AUROC for detecting significant stenosis, adequate assessment of the V1 segment is often not possible due to anatomic difficulties. Assessment of the V2 segment is feasible but yielded few stenoses. Hence, we consider usefulness of DUS for screening of extracranial vertebral artery stenosis limited.

## 1 INTRODUCTION

About 25% of all strokes are posterior circulation strokes (Bamford, Sandercock, Dennis, Burn, & Warlow, 1991; Bogousslavsky, van Melle, & Regli, 1988). Vertebrobasilar stenosis is an important cause of posterior circulation stroke and is found in 26.2% patients with such stroke (Marquardt, Kuker, Chandratheva, Geraghty, & Rothwell, 2009). In addition, the presence of vertebrobasilar stenosis is known to double or triple the risk of a recurrent posterior circulation stroke (Gulli, Khan, & Markus, 2009; Marquardt et al., 2009).

Digital subtraction angiography (DSA) is the gold standard for the diagnosis of vertebrobasilar stenosis. However, it is expensive and patients undergoing DSA have a 1–2% risk of neurological complications (Heiserman et al., 1994; Willinsky et al., 2003). Contrast‐Enhanced Magnetic Resonance Angiography (CE‐MRA), Computed Tomography Angiography (CTA) and Duplex UltraSonography (DUS) offer less invasive alternatives for imaging the vertebrobasilar system. Both CE‐MRA and CTA have high sensitivity and specificity for detecting vertebral artery stenosis (Khan, Cloud, Kerry, & Markus, 2007; Khan, Rich, Clifton, & Markus, 2009). However, they require the use of radiation and intravenous contrast material that have their own disadvantages. DUS is a cheap and noninvasive imaging technique that has proven to be reliable for diagnosing carotid stenosis (Golledge et al., 1999). For vertebrobasilar stenosis, however, its diagnostic value is less clear (Ackerstaff, Grosveld, Eikelboom, & Ludwig, 1988; de Bray et al., 2001; Hua et al., 2009; Khan et al., 2007, 2009). We investigated the feasibility of DUS as a screening tool for the detection of extracranial vertebral artery stenosis in patients with posterior circulation TIA or stroke and assessed its diagnostic characteristics in comparison with CTA.

## 2 MATERIALS & METHODS

### 2.1 Patients

We retrospectively retrieved data on all patients who underwent a DUS of the extracranial large arteries in the period 2008–2012 in the Department of Clinical Neurophysiology of a large teaching hospital. Of these patients, matching CTA studies were retrieved. Only patients who were diagnosed with a TIA or ischemic stroke of the posterior circulation were included. In total 342 patients fulfilled the inclusion criteria. If patients had more than one examination, only the first one was included. Formal approval from the local ethics committee was not indicated because this study was based on routinely collected data.

### 2.2 Duplex ultrasonography

DUS was performed by a qualified technician in clinical neurophysiology with a color‐coded duplex machine (iU22, Philips, Eindhoven, the Netherlands) equipped with a compound imaging 9–3 MHz linear‐array transducer. A vascular preset was used. Patients were investigated in a supine position with the neck slightly extended. The vertebral artery was localized in a longitudinal plane at the sixth cervical vertebra where the vertebral artery usually enters the transverse foramina. The diameter of the artery was measured. With doppler, direction of flow was established. For analysis, we divided the course of the vertebral artery into two segments: V1 (from the origin of the vertebral artery until the point where it enters the fifth or sixth cervical vertebra) and V2 (the part of the vertebral artery that courses cranially to the transverse foramina until it emerges besides the lateral mass of the atlas) (Cloud & Markus, 2003). Segments were studied in B mode and color mode. Doppler samples were taken and Peak Systolic Velocity (PSV) was recorded. Criteria used for grading ≥50% stenosis were focal elevated blood flow velocity with a PSV cut‐off point at the V1 segment of the vertebral artery of 140 cm/s and 125 cm/s at the V2 segment (Ackerstaff et al., 1984, 1988; Hua et al., 2009; Koch, Romano, Park, Amir, & Forteza, 2009).

### 2.3 CT angiography

CTA studies were performed on a 64 slice CT scanner (Lightspeed VCT; General Electric Medical Systems, Little Chalfont, Buckinghamshire, United Kingdom) with the gantry angled to the orbitomeatal line, 64 1‐s rotations of 1.25‐mm collimation, a table speed of 1.5 mm/s, a 512 × 512 matrix, a 16‐cm field of view, a tube voltage of 120 kV with a maximum tube current of 600 mA and a small focus. 50 cc Visipaque contrast material [320 mg iodine/mL] was injected intravenously at a rate of 6 cc/s with an automated power injector. A timing bolus was used to calculate the injection delay after contrast passage through the aortic arch for automated triggering of image acquisition, followed by a ‘chaser’ bolus of 20 cc saline. The CTA source image data were postprocessed creating coronal, axial, and sagittal source image reconstructions with a dedicated image processing computer workstation, after which luminal measurements were done with an automated vessel tracking software module (Advantage Workstation 4.4 & AVA Express; Global Electronics Medical Systems). Measurement of the degree of stenosis was done according to the NASCET criteria (North American Symptomatic Carotid Endarterectomy Trial (NASCET) Steering Committee, 1991). A vertebral artery stenosis of ≥50% was considered significant. Measurements were done by an experienced neuroradiologist (GL) and resident in neuroradiology (HH).

### 2.4 Analysis

For all included patients DUS and CTA studies were reevaluated. DUS reevaluation was done without knowledge of the CTA results and vice versa. CTA was used as “gold standard.” The results of the DUS were compared with the results from CTA. The results of the CTA were dichotomized in “no significant stenosis” and “significant stenosis.” For each segment (V1 and V2 segment) ROC curves were drawn and the area under the ROC curve (AUROC) was determined. Sensitivity and specificity of DUS were calculated, at first at the above mentioned established cut‐off values. As we aimed at studying whether DUS could be used as a screening tool for possible vertebral stenosis and hence as a selection tool for patients needing to undergo more invasive imaging with CTA, we calculated which DUS PSV cut‐off value resulted in highest sensitivity with acceptable specificity. In addition, we calculated positive predictive values (PPV) and negative predictive values (NPV) for DUS at the predefined cut‐off values.

## 3 RESULTS

A total of 425 patients who were diagnosed with a TIA or ischemic stroke of the posterior circulation had a DUS of the extracranial arteries; in 83 of them no CTA was made. From the remaining 342 patients, with 684 segments, 10 segments were occluded and hence excluded.

Therefore, 337 patients were included. Of these 198 patients were men (59%) and the median age was 67 years (range from 26 to 93). Table 1 shows an overview of all measured vertebral artery segments in our cohort.

**Table 1:** Vertebral artery stenosis as measured by DUS and CTA

		CT angiography			
		Stenosis	No stenosis	No measurement	Total
Duplex Ultrasonography	*V1 segment*				
	Stenosis (PSV>140 cm/s)	12	40	0	52
	No stenosis	19	307	0	326
	No measurement	29	201	66	296
	Total	60	548	66	674
	*V2 segment*				
	Stenosis (PSA>125 cm/s)	2	8	0	10
	No stenosis	2	656	5	663
	No measurement	0	1	0	1
	Total	4	665	5	674

DUS detected significant stenosis in 62 segments (6%), mostly at the V1 segment of the vertebral artery (52 segments, 84%). CTA detected 64 significant stenoses (5%) also mostly located at the first segment of the vertebral artery (60 segments, 94%). Of the 62 stenoses found with DUS, 14 (23%) were confirmed with CTA.

**Figure 1:**
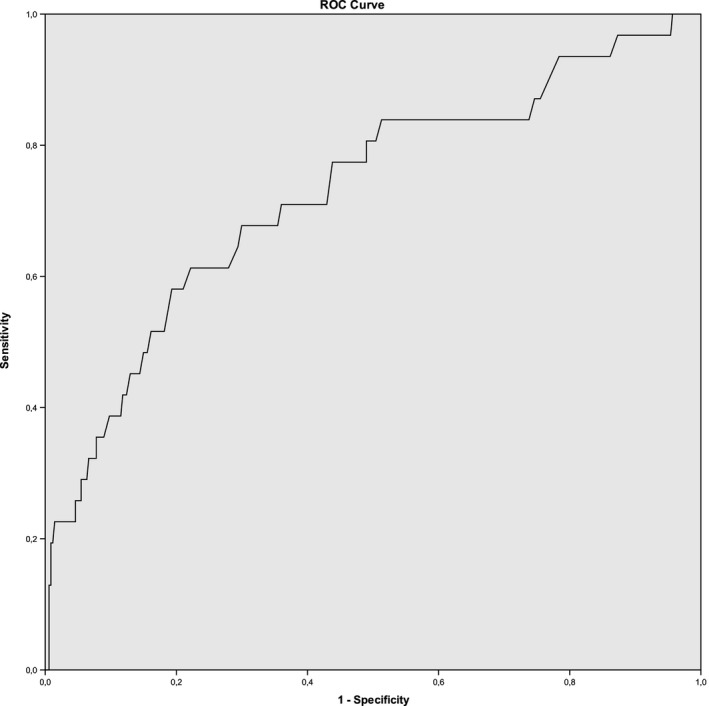
Receiver operating characteristic curve. Detection of significant vertebral artery stenosis (≥50%) on CTA by DUS based on peak systolic velocities (PSV) for the V1 segment of the vertebral artery

Of the 674 V1 segments, 608 segments (90%) could be adequately measured with CTA and 378 segments (56%) with DUS. DUS detected significant stenosis in 52 segments (14%) of which 12 were confirmed with CTA. The ROC curve showed that the PSV as measured by DUS was fairly capable of discriminating whether there was a vertebral artery stenosis at the V1 segment of this vertebral artery on CTA (AUROC 0.73, 95% CI: 0.63–0.83, Figure 1).

Of the 674 V2 segments, 669 segments (99%) could be measured adequately with CTA and 673 segments (100%) with DUS. DUS detected 10 stenoses (1%) at the V2 segment of which 2 were confirmed with CTA. Due to this low number of stenoses, reliable assessment of the diagnostic characteristics of DUS compared with CTA was considered not possible at the V2 segment.

Although we did not measure distal vertebral artery segments with DUS, we did search with CTA for distal vertebral artery stenosis and occlusions, PICA ending vertebral arteries and basilar artery stenosis and occlusion. We found 40 distal vertebral artery stenoses, 24 distal vertebral artery occlusions, 31 PICA ending vertebral arteries, 6 basilar artery stenoses and 4 basilar artery occlusions. In these arteries, four proximal vertebral artery segments (V1 and V2 segments) were found to have an occlusion according to DUS. None of these were confirmed with CTA (webtable, Table S1).

Table 2 shows the sensitivities and specificities for the various cut‐off values of the PSVs at the V1 segment of the vertebral artery. If calculated according the predefined cut‐off of 140 cm/s, the sensitivity of DUS was 39% and the specificity was 88% with a corresponding positive predictive value (PPV) of 23% and the negative predictive value (NPV) of 94%. A cut‐off at PSV of 90 cm/s at the V1 segment resulted in best sensitivity with acceptable specificity. Prior chance of stenosis of 8.2% (31/378) reduces to a chance of 3.0% (5/169) if DUS shows a PSV < 90 cm/s. Also NPV improved from 94% at a cut‐off of 140 cm/s to 97% at a PSV cut‐off of 90 cm/s. For the V2 segment such an analysis was not feasible because of the limited number of stenoses.

**Table 2:** Sensitivity and specificity for cut‐off values of peak systolic velocity on duplex ultrasonography for significant vertebral artery stenosis as measured by CT angiography

CT‐angiography	V1 Segment		
	PSV cut‐off value (cm/s)	Sensitivity (%)	Specificity (%)
Stenosis ≥ 50%	140	39	88
	130	48	85
	120	61	78
	110	68	69
	100	71	57
	90	84	47
	80	84	37
	70	87	25

## 4 DISCUSSION

In this study, we found that DUS was a fairly adequate test for detecting vertebral artery stenosis at the V1 segment (AUROC 0.73, 95% CI: 0.63–0.83). However, in almost half of all measured V1 segments no adequate PSV could be obtained due to technical difficulties such as the often deep and posterior origin of the vertebral arteries, calcified lesions, a tortuous course, or short neck stature. At the V2 segment few stenoses were found and therefore we could not perform reliable analysis for this segment. Hence, we think that the usefulness of DUS in diagnosing extracranial vertebral stenosis is limited.

Compared with previous studies in patients with posterior stroke, we found approximately the same prevalence of significant vertebral artery stenosis with CTA (Caplan et al., 2004; Marquardt et al., 2009). However, we were not able to detect most of these stenoses with DUS. This resulted in lower sensitivity of DUS compared with earlier studies (de Bray et al., 2001; Hua et al., 2009). In addition, we found that our proportion of adequate visualization of the V1 segment was rather low. Most studies report that the V1 segment is less accessible for DUS but nevertheless report higher frequencies of adequate visualization (72–87%) than we achieved (Ackerstaff et al., 1988; Hallerstam & Rosfors, 2004; Matula, Trattnig, Tschabitscher, Day, & Koos, 1997).

On the basis of our results we found a PSV of 90 cm/s to be the optimal cut‐off value for detection of a stenosis at segment 1 in the vertebral artery. Previous studies mostly recommended higher cut‐off values (Hua et al., 2009; Škoda, Kalvach, Procházka, & Svárovský, 2014; North American Symptomatic Carotid Endarterectomy Trial (NASCET) Steering Committee, 1991). However, our aim was to study at what cut‐off value sensitivity was highest with acceptable specificity (to prevent false negatives) instead of finding an optimal cut‐off value with the highest combination of both acceptable sensitivity and specificity. With this cut‐off value the prior chance of vertebral artery stenosis at the V1 segment is reduced from 8.2% to 3.0%.

This study has several limitations. First, we collected all data retrospectively which might reduce the generalizability of our results. However, in our clinic, all stroke and TIA patients are treated according to a uniform stroke protocol that includes a DUS of the extracranial large arteries. Hence, we think that out cohort represents a quite complete cohort of all patients that suffered from a posterior circulation stroke or TIA in the predefined period. Second, we used CTA as “gold standard” for detecting vertebral artery stenosis instead of DSA. This was done for pragmatic reasons, most stroke patients do not routinely undergo DSA (i.e., because of complication risks). In addition, previous studies show that CTA is adequately capable of detecting vertebral artery stenosis (Farres et al., 1996; Khan et al., 2009). Third, the cause of posterior circulation stroke or TIA might be thrombo‐embolic instead of hemodynamic. For this, evaluation of plaque morphology would be necessary which is rather difficult with ultrasound in the vertebral arteries. In addition, our study focus was on the usefulness of DUS in establishing significant vertebral artery stenosis instead of plaque morphology in case of vertebral artery stenosis.

Our results are rather disappointing with regards to the accuracy of DUS in detecting vertebral artery stenosis. Almost half of all vertebral artery segments could not be assessed with DUS. As the quality of DUS does very much depend on the experience of the ultrasound technician one could argue that our results could be due to lack of experience of our ultrasound technicians. However, our ultrasound technicians are highly qualified in clinical neurophysiology and perform several DUS studies every day.

**Figure 2:**
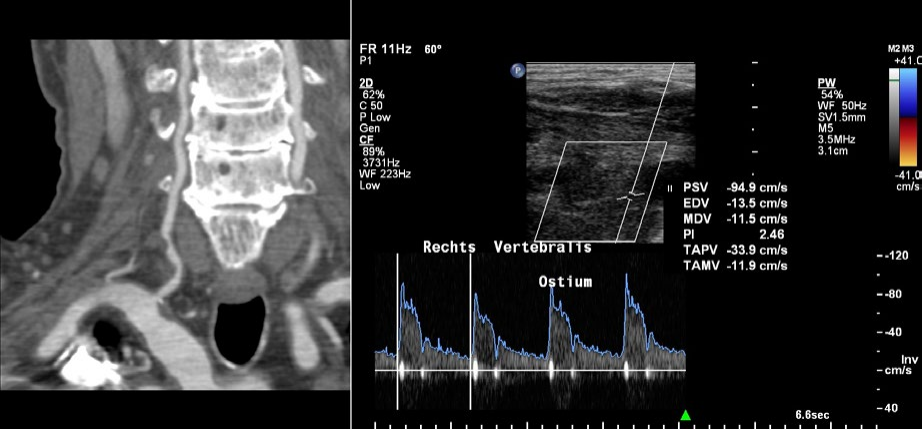
The CT angiography (right) shows a stenosis at the V1 segment. With DUS (left) a normal PSV is found

Another explanation for our results might be that at median age of 67 years many patients may have degenerative changes of the cervical vertebrae which might hinder adequate assessment of the vertebral arteries. However, if adequate assessment of the vertebral artery was possible, DUS was not able to detect stenosis in most cases. A possible explanation might be that the PSV may erroneously seem normal as, for instance, the stenosis is localized more distally (Figure 2). One could argue that the end‐diastolic velocity, the B‐mode image and certain spectral changes should also be studied in addition to PSV resulting in a more reliable assessment of the vertebral artery with DUS. However, previous studies suggest that PSV is the most accurate predictor of stenosis in the extracranial vertebral artery (Hua et al., 2009; North American Symptomatic Carotid Endarterectomy Trial (NASCET) Steering Committee, 1991).

## 5 CONCLUSIONS

Our data show that assessment of the vertebral artery is difficult, especially at the V1 segment. This is, however, the segment most prone to atherosclerotic changes. In addition, if assessment of the vertebral artery with DUS was possible, there was an adequate detection of stenosis in only the minority of patients. Hence, the usefulness of the DUS as a screening tool for extracranial vertebral artery stenosis seems to be limited.

## CONFLICT OF INTEREST

On behalf of all authors, the corresponding author states that there is no conflict of interest.
