# Supplementary material for: Duplex ultrasonography for the detection of vertebral artery stenosis: A comparison with CT angiography
Source: Brain Behav. 2017 Jun 29;7(8):e00750. doi: 10.1002/brb3.750 (PMC5561311; doi:10.1002/brb3.750)
Supplement: Supplementary file 1 [file BRB3-7-e00750-s001.docx]

|  | N | Adequate DUS  Measurements | DUS occlusion | CTA occlusion |
| --- | --- | --- | --- | --- |
| **Distal VA stenosis** |  |  |  |  |
| *Right side* | *14* |  |  |  |
| VA segment 1 |  | 9 | 1 | 0 |
| VA segment 2 |  | 14 | 0 | 0 |
| *Left side* | *26* |  |  |  |
| VA segment 1 |  | 13 | 0 | 0 |
| VA segment 2 |  | 25 | 1^*^ | 1^*^ |
| **Distal VA occlusion** |  |  |  |  |
| *Right side* | *18* |  |  |  |
| VA segment 1 |  | 6 | 0 | 2 |
| VA segment 2 |  | 13 | 0 | 1 |
| *Left side* | *6* |  |  |  |
| VA segment 1 |  | 1 | 0 | 0 |
| VA segment 2 |  | 5 | 0 | 0 |
| **PICA ending VA** |  |  |  |  |
| *Right side* | *18* |  |  |  |
| VA segment 1 |  | 9 | 1 | 0 |
| VA segment 2 |  | 18 | 0 | 0 |
| *Left side* | *13* |  |  |  |
| VA segment 1 |  | 8 | 1^*^ | 1^*^ |
| VA segment 2 |  | 13 | 0 | 0 |
| **Basilar Artery stenosis** | *6^#^* |  |  |  |
| VA segment 1 |  | 5 | 0 | 1 |
| VA segment 2 |  | 12 | 0 | 0 |
| **Basilar Artery occlusion** | *4^#^* |  |  |  |
| VA segment 1 |  | 1 | 0 | 0 |
| VA segment 2 |  | 8 | 0 | 0 |

DUS = Duplex Ultrasonography; CTA = CT angiography; VA = Vertebral Artery; PICA = Posterior Inferior Cerebellar Artery.

*not corresponding

# every basilar artery originates from 2 vertebral arteries
